# Dysphagia lusoria: A vascular etiology?

**DOI:** 10.1002/jgh3.12366

**Published:** 2020-05-28

**Authors:** Michael Coles, Victoria M. Madray, Chinmaya Mareddy, Deepak Kapoor, Amol Sharma

**Affiliations:** ^1^ Augusta University Health Medical Center Augusta Georgia USA

**Keywords:** aberrant right subclavian artery, esophageal motility disorders, lower esophageal sphincter, manometry

## Abstract

Dysphagia lusoria is difficulty swallowing as a result of extrinsic esophageal compression by an aberrant right subclavian artery (ARSA). We present the case of a 59‐year‐old patient with ARSA and history of chronic dysphagia. Vascular decompressive surgery was performed, but it failed to resolve his symptoms. Esophageal manometry indicated concomitant esophageal gastric junction outflow obstruction in the setting of a small hiatal hernia. Our case highlights the diagnostic dilemma surrounding dysphagia lusoria and identification of cases that should undergo surgical repair. Based on a thorough review of the literature and our case, we propose a complete foregut workup for possible other causes as potential etiologies of dysphagia prior to surgical treatment of dysphagia lusoria.

## Graphical abstract

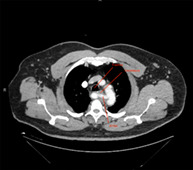

Dysphagia lusoria is difficulty swallowing as a result of extrinsic esophageal compression by an aberrant right subclavian artery (ARSA). Based on a thorough review of the literature and our case, we propose a complete foregut workup for possible other causes as potential etiologies of dysphagia prior to surgical treatment.

## Introduction

Dysphagia lusoria is a rare embryologic defect of the aortic arch vasculature characterized by an aberrant retroesophageal course of the right subclavian artery (RSA), comprising a vascular sling. This may manifest clinically with symptoms of dysphagia or reflux.^1^ The anomaly most commonly involves the right subclavian artery (0.5–2.0% of the population) or, rarely, the left subclavian artery (LSA) when there is a right‐sided aortic arch (0.05–0.1%) and left‐sided ligamentum arteriosum.^2^ The latter constitutes a true vascular *ring*, which typically constricts the esophagus and trachea more significantly when compared with an aberrant RSA, which is classified as a noncircumferential vascular *sling*.^3^ Surgical vascular decompression is often used to rectify the aberrant vascular morphology. However, less than half of aberrant arteries, an estimated 20–40%, result in trachea–esophageal symptoms.^4^ Based on our case, we recommend a thorough evaluation of other potential foregut etiologies of dysphagia lusoria prior to vascular repair.

### *Case report*

A 59‐year‐old African American male with a history of hypertension, tobacco abuse, and obstructive sleep apnea presented to the hospital with symptoms of unstable angina of a duration of 3 months. Investigations were suggestive of a non‐ST‐elevation myocardial infarction (NSTEMI); he subsequently underwent left heart catheterization (LHC), with coronary angiography revealing 99% stenosis of the right coronary. An additional finding was the presence of a vascular anomaly in the form of an aberrant RSA, which arose distal to the origin of the LSA and followed a retroesophageal course.

LHC was complicated by knotting of the guide catheter and nonflow, limiting RSA dissection during knot retrieval. At follow‐up, the patient continued to report nonexertional chest discomfort, in addition to continuing dysphagia and dyspnea. His dysphagia symptoms were largely overlooked during initial presentation given the acuity of his NSTEMI. However, upon further questioning, he reported progressive dysphagia to solid foods for 3 months, multiple times a week; “stuck” food bolus sensation; and occasional emesis. He denied alarm symptoms.

**Figure 1:**
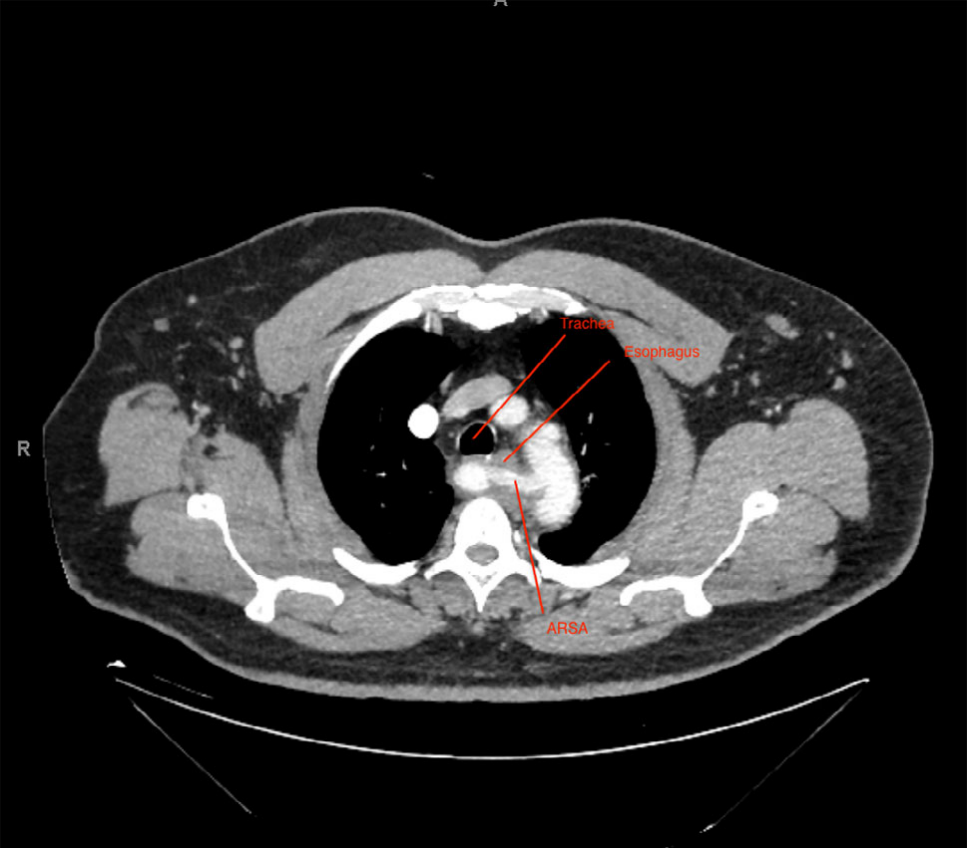
Computed tomography angiography without oral contrast demonstrating the retroesophageal course of the aberrant right subclavian artery in the sagittal plane.

The patient's symptomatology was highly suspicious for dysphagia lusoria given the aberrant RSA. Follow‐up computed tomography angiography (CTA) without oral contrast was performed, which confirmed retroesophageal course of the RSA and mass effect on the posterior aspect of the upper esophagus (Fig. 1), as well as mild to moderate anterior displacement of the trachea. The patient was offered elective surgical reimplantation of the RSA in the right carotid artery, to be performed 3 months post‐NSTEMI. In the interim, the patient underwent a diagnostic esophagogastroduodenoscopy (EGD) to rule out other intraluminal etiologies of dysphagia. EGD demonstrated possible extrinsic compression seen in the proximal esophagus with normal esophageal mucosa.

During the interim, the patient experienced worsening symptoms of both dysphagia and nonexertional chest discomfort. This was concerning because of a possible hematoma secondary to RSA dissection, which was noted during attempted guide catheter extraction at the previous LHC. After unremarkable repeat angiography, the patient underwent detachment and ligation of the RSA from its aortic origin, along with right carotid–RSA bypass using an 8‐mm interposition Dacron graft.

The patient reported improvement following this procedure; however, dysphagia recurred within 6 months despite proton pump inhibitor therapy, intentional weight loss, and cessation of smoking and alcohol intake. An esophageal manometry demonstrated EGJ outflow obstruction in the setting of a 2‐cm hiatal hernia. Workup with esophagram and pH study was pending, but the patient was lost to follow‐up.

## Discussion

We present a unique patient with suspected dysphagia lusoria in the setting of confirmed aberrant RSA, who underwent surgical repair without improvement of symptoms. Surgical repair was followed by EGD but not by other investigations such as high‐resolution esophageal manometry (HREM), barium esophagram, and various esophageal pH studies. There were discordant findings of a small hiatal hernia on HREM, not appreciated on EGD. While not sensitive for detecting hiatal hernia, if detected by HREM, it is very specific.^5^ Patients with hiatal hernia demonstrate a higher extent of reflux, prolonged acid clearance, and impaired esophageal emptying, which may result in associated symptoms of dysphagia and noncardiac chest pain.^6^, ^7^, ^8^ This case illustrates two important lessons: First, it highlights the need for a thorough workup with esophageal function testing to assess for coexisting causes of dysphagia. Second, aberrant RSA with associated dysphagia lusoria may be a cause of dysphagia, but less than half the time. In the majority of aberrant RSA cases, a highly constrictive, circumferential, vascular ring does not impinge on the esophagus enough to cause dysphagia. Comprehensive evaluation and management should precede surgical repair of aberrant RSA in only refractory cases of suspected dysphagia lusoria. Care for such patients should occur in a multidisciplinary manner, involving cardiologists, gastroenterologists, and vascular surgeons.

A published case study followed six patients with aberrant RSA and concomitant unspecified esophageal dysmotility. Three of the six patients responded to medical therapy alone, and the remaining three patients underwent vascular decompression surgery for persistent symptoms. Of those who underwent surgical repair, two of the three surgical patients had resolution of their symptoms.^9^ Assessing the therapeutic response to vascular decompression may be challenging, especially in the presence of concomitant esophageal dysmotility. In our patient, the vascular procedure was premature and clearly not effective in achieving symptom relief.

In conclusion, we propose that the diagnostic workup and treatment priority in dysphagia lusoria should first focus on investigating for an underlying hiatal hernia or esophageal motility disorder. Validation of this care pathway is reinforced by the substantial risk of treatment failure, as well as the morbidity and even mortality risk incurred by RSA–carotid bypass. Rare cases of marked aneurysmal dilation of the aberrant RSA and/or the presence of a bicarotid (so‐called bovine) trunk with or without dysphagia would favor vascular repair due to the elevated risk of rupture and potential severe esophageal compression.^0010^
